# Tablets Made from Paper—An Industrially Feasible Approach

**DOI:** 10.3390/ph15101188

**Published:** 2022-09-26

**Authors:** Ayat Abdelkader, Christoph Moos, Adrien Pelloux, Marcus Pfeiffer, Christian Alter, Stefan Kolling, Cornelia M. Keck

**Affiliations:** 1 Department of Pharmaceutics and Biopharmaceutics, Philipps-Universität Marburg, Robert-Koch-Str. 4, 35037 Marburg, Germany; 2 Assiut International Center of Nanomedicine, Al-Rajhy Liver Hospital, Assiut University, Assiut 71515, Egypt; 3 Institute of Mechanics and Materials, Technische Hochschule Mittelhessen, Wiesenstr. 14, 35390 Giessen, Germany; 4 MEDELPHARM, Science Lab, Rue du Chat Botté 615, 01700 Beynost, France

**Keywords:** paper, granules, oral drug delivery, tablet manufacturing, mechanical modeling, instrumented die compression

## Abstract

Many orally administrated drugs exhibit poor bioavailability due to their limited solubility. The smartFilm technology is an innovative approach to improve the drug aqueous solubility, where the drug is embedded within the matrix of cellulose-based paper in an amorphous state, hence increasing its solubility. Despite its proven effectiveness, smartFilms, i.e., pieces of paper, exhibit limited flowability and are not easy to swallow, and thus oral administration is not convenient. In addition, there is a lack of knowledge of their mechanical behavior under compression. This study aimed to transform unloaded smartFilms, i.e., paper, into a flowable physical form and investigated its mechanical behavior when compressed. Granules made of paper were prepared via wet granulation and were compressed into tablets. The influence of using different amounts and forms of sucrose, as a binder, on the pharmaceutical properties of the produced granules and tablets was studied and the most suitable composition was identified by using instrumented die experiments. For this, the Poisson’s ratio and Young’s modulus were determined for different compaction force levels and the deformation behavior was estimated with the Heckel mathematical model. All granule batches showed good flowability with angle of repose values between 25–35°. Granule batches with ≤30% dry sucrose content produced tablets that fulfilled the European Pharmacopeia requirements, and the compaction behavior of the granules was found to be comparable to the behavior of classical binders and compression enhancers. Paper can be transferred into granules. These granules can be used as suitable intermediate products for the production of tablets made of paper in large, industrial scale.

## 1. Introduction

Oral administration is the most common route of drug delivery, with advantages including high patient compliance, low risk of infection and minimal sterility constraints, which simplifies the production process and reduces costs [1]. Efficient oral delivery of various active pharmaceutical ingredients (API) is extremely influenced by their physicochemical and/or biopharmaceutical properties, e.g., solubility, chemical stability, intestinal permeability, metabolic stability, etc.

Recently, smartFilms were introduced as a novel oral drug delivery system that can incorporate API in an amorphous state within the matrix of cellulose-based paper, leading to the improvement of the dissolution rate and the kinetic solubility of the API [2]. SmartFilms can be obtained by dissolving a poorly soluble API into a suitable solvent and by adding this solution to a cellulose-based matrix, i.e., paper. After drying, the paper, i.e., the resulting “smartFilm”, contains the API in amorphous state, resulting in an enhanced dissolution rate and kinetic solubility of the API [2,3]. Despite being a promising oral drug delivery system, the administration of paper sheets (i.e., smartFilms) is not convenient for the patient. Due to this, the transfer of the smartFilms into appropriate oral dosage forms is important for improving patient compliance. Several studies already reported the successful compression of drug-loaded smartFilms into tablets without the addition of any excipients and demonstrated that the produced tablets fulfilled all requirements of the European Pharmacopeia [4,5,6].

Nevertheless, paper sheets and smartFilms in their original state exhibit inadequate flowability, rendering them unsuitable candidates for high-speed tablet manufacturing and large scale tablet production [4]. In light of this, the transformation of smartFilms into a free-flowing physical form along with understanding their mechanical behavior under compression is a major prerequisite for a wider exploitation of the smartFilm technology, because it opens the possibility to produce smartFilm tablets on an industrial, large scale.

Granulation is a key processing step in the production of many pharmaceutical tablets [7]. In comparison to raw powders, granules exhibit enhanced flowability and compaction characteristics, and thus can be compressed easily into tablets with a uniform API content [7]. Consequently, the transformation of paper and/or smartFilms into granules was expected to improve their limited flowability.

Preliminary data, that produced granules from paper without API (i.e., unloaded paper granules) without further excipients while using purified water as binder, could already prove this assumption but showed that the resulting granules possess a high elasticity. The high elasticity of the granules proved to be a non-suitable characteristic for the production of tablets in continuous mode, because during the production, the granules jumped out of the die. In addition, the resulting tablets were too soft and could not fulfil the requirements of the European Pharmacopeia [8]. Therefore, to decrease the elasticity and to increase the plastic deformation of the paper-based granules, it was hypothesised that the addition of sucrose might improve the compressibility of the paper granules. The aim of this study was to prove or disprove this theory and to identify the most suitable sucrose contents and the most suitable production process for granules made of paper that can be used for the production of smartFilm tables in large, industrial scale.

Sucrose can be used as a dry binder, i.e., prior to granulation, it is added as dry powder to the other ingredients of the tablet. However, it can also be utilized as a liquid binder. In this case, aqueous sucrose solutions, containing 50–67% (*w*/*w*) sucrose, are used as granulation liquid for the granulation of the powdered ingredients of the tablet [9]. In order to identify the most suitable process, granules made of paper were produced with different sucrose contents and by using sucrose as either dry binder (batches B2–B6, Table 1) or liquid binder (batches B7–B11, Table 1), respectively. B1 granules were composed of paper without sucrose and served as benchmark control. B2–B6 granules were produced while using sucrose as dry binder and B7-B11 granules were produced while using sucrose as liquid binder, i.e., aqueous solutions of sucrose. The sucrose content was varied from 10% to 50%, respectively (Table 1). 

After the production of the granules, size, shape and the pharmaceutical properties according to the European Pharmacopeia, i.e., bulk density, tapped density, Hausner ratio, Carr index and angle of repose were determined [10]. In the next step, the paper granules were used for the production of tablets from which the pharmaceutical properties according to the European Pharmacopeia were determined. 

In addition, the mechanical behavior of paper-based granules under compression was investigated and compared to the properties of conventional binders. 

Mechanical properties, such as elastic and plastic behaviors, play important roles for the understanding of the granule’s mechanical behavior under compression. In addition, mechanical characterization is also pivotal for the simulation modeling of pharmaceutical materials (e.g., granules) under compression. The use of simulation modelling in pharmaceutical industry is becoming not only important to improve the quality of the product but can also be used to understand the influence of materials and processes on the final pharmaceutical product properties. In addition, it can also help to reduce the productions costs [11]. Therefore, in the second step of this study, instrumented die compression tests were performed to characterize the mechanical behaviour of the paper granules under compression [12]. 

## 2. Results and Discussion

### 2.1. Preparation and Characterization of Paper Granules

**Figure 1:**
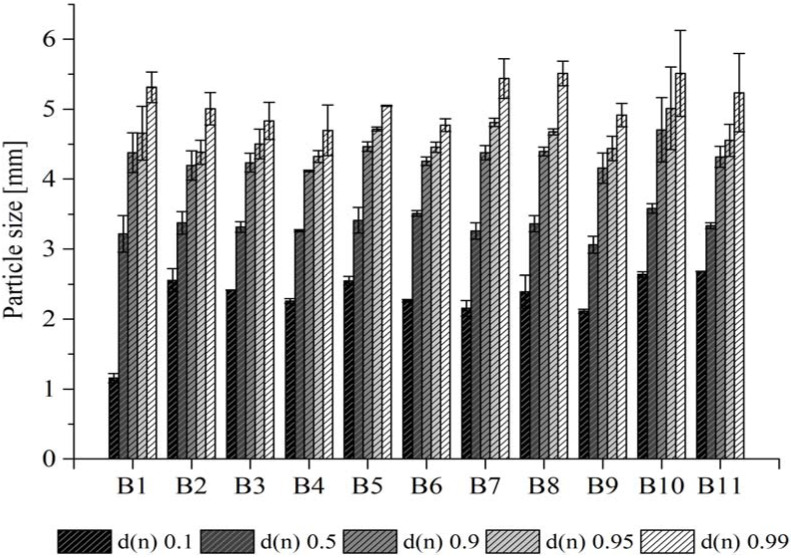
Numeric particle size distribution of paper granules from different batches based on the predetermined Feret’s diameter. A d(n) 0.5 represents the size where 50% of all granules are smaller or equal to the given number.

The paper granules without sucrose possessed a particle size of 3 mm (Table 2). The addition of sucrose resulted in slightly larger sizes. No significant differences (*p* > 0.05) were observed between the batches where sucrose was used as dry or liquid binder (Table 2). Figure 1 shows the numeric particle size distributions of the different paper granules. The results indicate similar d(n) 0.9, d(n) 0.95 and d(n) 0.99 values for all the prepared granules either with or without sucrose, with a slight trend towards lager sizes for the granules that were prepared with the liquid sucrose binder. An aspect ratio in the range of 1.38–1.52 was observed for all the prepared granules either with or without sucrose. According to literature, a value of 1.2 is considered to present spherical particles. With this, data indicate that the paper granules possess a slightly elongated shape [13].

Bulk and tapped density of B2–B6 increased significantly (*p* < 0.01) as the amount of sucrose increased. On the other hand, B7–B11 exhibited an inconsistent increase in terms of bulk and tapped density, especially in case of sucrose aqueous solutions of high concentration (Table 2). This can be attributed to the high viscosity of such solutions which might result in an inhomogeneous distribution of the granulation liquid among the formed granules, hence affecting their properties. Hausner’s ratio and Carr’s index were used as an indication of the flowability of the granules. Table 2 shows that Hausner’s ratio and Carr’s index values were in the range from 1.12 to 1.18 and from 11 to 15.0, respectively, which indicates good flowability and compressibility of the prepared granules according to the European Pharmacopeia [10].

Regarding the angle of repose, B1 (no sucrose) exhibited a value in the range of 41–45°. This indicates only passable flowability and that the produced granules might hang up inside the hopper during tablet manufacturing. Indeed, the adherence of the granules to the hopper was recognized during the production of the tablets and it was attributed to the electrostatic effects of paper [14]. The addition of sucrose decreased the angle of repose. B3, B4, B8 and B9 showed values in the range of 31–35°, which suggests good flowability behavior. B5, B6, B10 and B11 exhibited values in the range of 25–30° (Table 2), which indicate excellent flowability according to the European Pharmacopeia [10]. Interestingly, the electrostatic adherence of the granules to the hopper disappeared when sucrose was added to the granules. The effects can be a superposition of many different effects [15]. In this study the reduced adherence can be for example due to an increase in size, rigidity and/or density and more research is needed to understand the effects in detail. In spite of this, the obtained results suggest that the die filling process using B2–B11 during high-speed tablet manufacturing will run efficiently with no major complications (e.g., without variations in tablet weight due to inconsistent die filling) [16].

### 2.2. Preparation and Characterization of Paper-Based Tablets Using the Produced Granules

**Figure 2:**
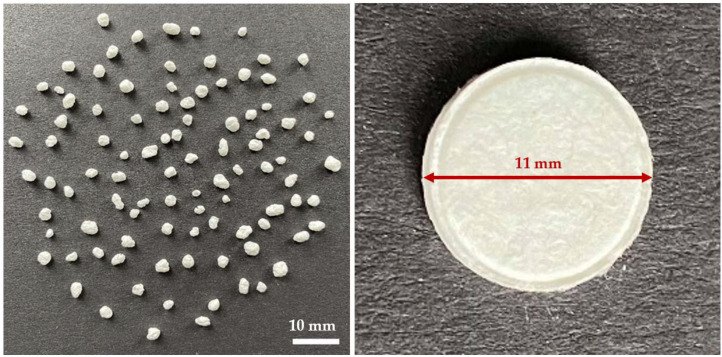
Paper granules with 20% sucrose content (B3) and the produced tablet made from the B3 paper granules.

In the next step, paper-based tablets were produced from all different paper granule batches. All sucrose containing granules led to smooth, slightly porous, and pale tablets (Figure 2). The manufactured tablets were then evaluated for their thickness, mass uniformity, friability, hardness and disintegration time, and the obtained data are summarized in Table 3. 

Regarding the thickness of the tablets, it was observed that the thickness of the produced tablets from the B1 batch was significantly higher (*p* < 0.01) when compared to the tablets produced from granule batches with the lowest sucrose content (B2 and B7). This indicates that tablets made from paper are less compact in the absence of a binder. The effect was expected, as the B1 batch tablets contained no sucrose and thus they were expected to possess the highest elasticity. The high elasticity of the material causes a stronger elastic relaxation of the tablet after the compression and, thus results in a higher thickness of the tablets. Moreover, it was found that the tablet thickness increased (*p* < 0.01) with increasing sucrose content (Table 3).

The tablet masses increased with increasing amounts of sucrose and were in the range between 172 and 283 mg. According to the criteria of the European Pharmacopeia, tablets fulfil the criteria of mass uniformity if not more than 2 of the individual masses (out of 20) deviate from the average mass by (i) more than 7.5% if the tablet mass of the tablets is in the range between 80–250 mg and by (ii) more than 5% if the tablet mass of the tablets is ≥80–250 mg [10].

The weight of the tablets produced from batches B1-B5 and B7-B10, i.e., tablets containing 0–40% sucrose, was <250 mg and all tablets fulfilled the criteria of the European Pharmacopeia. In contrast, tablets produced from batches B6 and B11, i.e., tablets that contained 50% sucrose, weighted more than 250 mg and 7 out of 20 tablets exceeded the 5% limit. Accordingly, the tablets produced from batches B6 and B11, i.e., tablets from paper granules with very high sucrose content (50%), could not fulfil the criteria (Table 3).

The friability of the produced tablets from all batches fulfilled the criteria according to the European Pharmacopeia, as a weight loss of less than 1.0% was observed for all tablets (Table 3). Such results suggest that all the produced tablets attain a sufficient mechanical strength to withstand handling and shipping. 

Regarding the resistance to crushing, the produced tablets from B1 were extremely fragile and exhibited very low values for the crushing strength (i.e., 20.81 ± 7.57), indicating low compaction of the tablets in the absence of sucrose (Table 3). On the other hand, the remaining batches, prepared with sucrose as a binder, produced tablets with higher values for the crushing strength. In addition, the produced tablets from batches B2–B6 or B7–B11 exhibited a significant increase in the crushing strength (*p* < 0.01) with increasing the sucrose content due greater deformation and better bonding between granules [17]. Such changes in tablets hardness are extremely important as they might affect disintegration behavior, the dissolution profile and bioavailability of the incorporated drug, hence its therapeutic efficacy [18].

The disintegration time of tablets produced from B1 was extremely rapid (i.e., 10 s, Table 3), which is consistent with previously reported data that indicated that such disintegration behavior might hinder the administration process of paper-based tablets and decrease patient compliance [4]. In contrast, the remaining batches, prepared with sucrose as a binder, produced tablets with a slower disintegration time. Moreover, the produced tablets from batches B2–B6 or B7–B11 showed a significant increase in the disintegration time (*p* < 0.01) with increasing the sucrose content. These results are consistent with the crushing strength data, indicating that harder tablets attain less porosity, and thus experience a slower disintegration. Furthermore, all the tablets that were prepared from batches B1–B4 fulfilled the criteria according to the European Pharmacopeia, as all tablets disintegrated within 15 min. On the other hand, all of the tablets that were prepared from batches B5 and B6 failed the criteria according to the European Pharmacopeia for uncoated tablets, as they did not disintegrate within 15 min. Tablets produced from batches B5 and B6 attain higher crushing strength values in comparison to batches B1–B4, which might be attributed to the presence of higher amounts of sucrose (i.e., 40% or 50%), which slows down the disintegration. It was also observed that using sucrose aqueous solution as a granulation liquid in the manufacturing of B7-B11 resulted in the production of rigid tablets that failed to disintegrate within 15 min. Solutions of binders are usually used in tablet production [19]. Disintegration of the tablets depends on many factors including the compression force, nature of the binder, method of tableting and mechanism of tablet disintegration [19]. All of these parameters were kept constant during the production of all tablets, so it can be assumed that the incorporation method of the binder (i.e., the solid state or the aqueous solution) was responsible for the observed increase in disintegration time.

Based on the data, it can be summarized that the addition of sucrose in dry form is most suitable, as it resulted in granules that can be compressed into tablets with good pharmaceutical profile. Paper granules with sucrose contents in the range between 20–30% (*w*/*w*) were identified to result in paper-based tablets with optimal pharmaceutical properties.

### 2.3. Mechanical Behaviour of Paper Granules under Compression

The evaluation of the mechanical behavior of paper granules under compression was carried out in the next part of the study by using three selected granule batches. B1 was selected to understand the mechanical behavior of granules in absence of a binder. B3 was chosen as it was able to produce paper-based tablets with an excellent pharmaceutical profile (i.e., optimum disintegration behavior). B6 was selected as a representative of paper-based tablets prepared using granules with a high sucrose content. 

**Figure 3:**
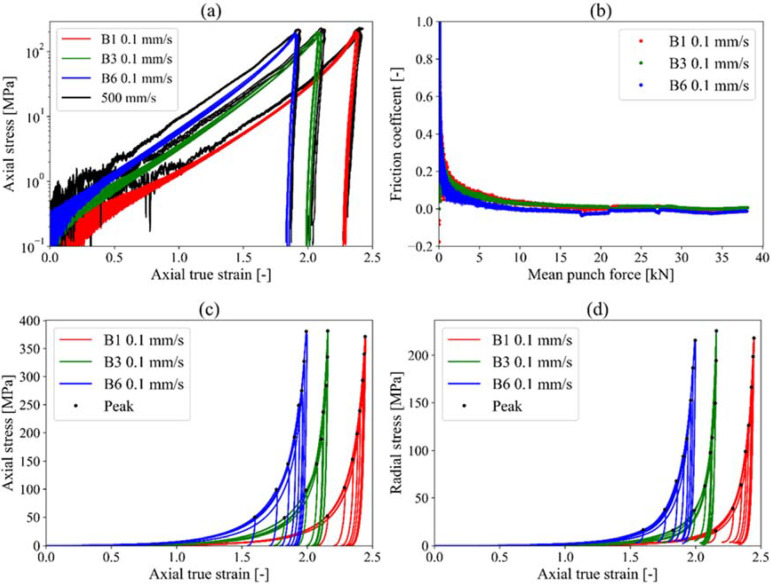
Experimental results of the instrumented die compression test of granules batches B1, B3 and B6. Strain rate dependency in logarithmic scale axial stress—axial true strain (**a**), friction coefficient—mean punch force (**b**), typical loading-unloading curves of granules compaction process indicating the influence of the sucrose content on axial stress—axial true strain (**c**) and radial stress—axial true strain (**d**) at 0.1 mm/s velocity.

The instrumented die compression tests were performed in three steps. In the first step, single compression experiments were performed by applying low and high compression velocities to the different formulations (Figure 3a). 

The results indicated that the compression velocity has only limited effects on the material behavior, i.e., strain rate effects are assumed to be negligible. Based in these findings, all other experiments were continued with one constant compression velocity (0.1 mm/s). The next step investigated the influence of the sucrose content within the granules on the die wall friction coefficient (Figure 3b). For all three formulations, i.e., the granules made of paper without sucrose (B1) and granules with medium (B3) and high sucrose content (B6), the friction coefficient was low for all of the tests. With this, it can be concluded that the compression of paper granules does not cause a remarkable friction between the granules and the wall of the die. In addition, as no significant differences were found between the friction coefficients from the different granules, it can be concluded that the influence of the sucrose content on the friction coefficient is neglectable.

The next step was the assessment of the material behavior during compression, i.e., the influence of the axial true strain on the axial stress and the radial stress, respectively (Figure 3c,d). Tests were performed within the press machine limits of 5 to 40 kN with compression force steps of 5 kN. Results show that an increase in sucrose content causes an increase in the stiffness of the material along with achieving a similar stress level at lower strains. Hence, it can be assumed that an increase in sucrose causes an increase in plastic hardening since powder compression is mainly due to plastic deformation. The effect is considered to occur due to the higher density that is obtained for the paper granules with higher sucrose content.

The higher density of the granules with higher sucrose content was confirmed by the decrease in the die filling height of the mean curves, which was 18.6 mm for B1, 14.44 mm for B3 and 11.75 mm for B6, respectively. Consequently, an increase in sucrose content caused not only a higher density but also decreased the porosity. Prior to the compression, the measured true density was 1.56 kg/m³ for all three batches, which is comparable to the true density of microcrystalline cellulose [20]. The mean curves resulting porosity of the die filling before compression was 0.914 for the batch without sucrose (B1), 0.889 for the batch with medium sucrose content (B3) and 0.864 for the batch with high sucrose content (B6).

Furthermore, the results also indicated that the compaction behavior of paper granules is comparable to the behavior of industrial powder under compression [20,21]. The compression of powder into tablets is generally divided into different stages [21,22]. The first stage is a rearrangement of the paper granules. In this stage, the rearrangement contributes most to the overall stress and hence the axial measured axial true strain is low. In the second step, an increasing densification of the granules takes place, hence, elastic, and plastic deformation of the granules becomes more dominant, which leads to an exponential increase in the stress—axial true strain curves. During the third stage, the unloading process, a nonlinear response of the stress—axial true strain can be observed (Figure 3c,d). 

**Figure 4:**
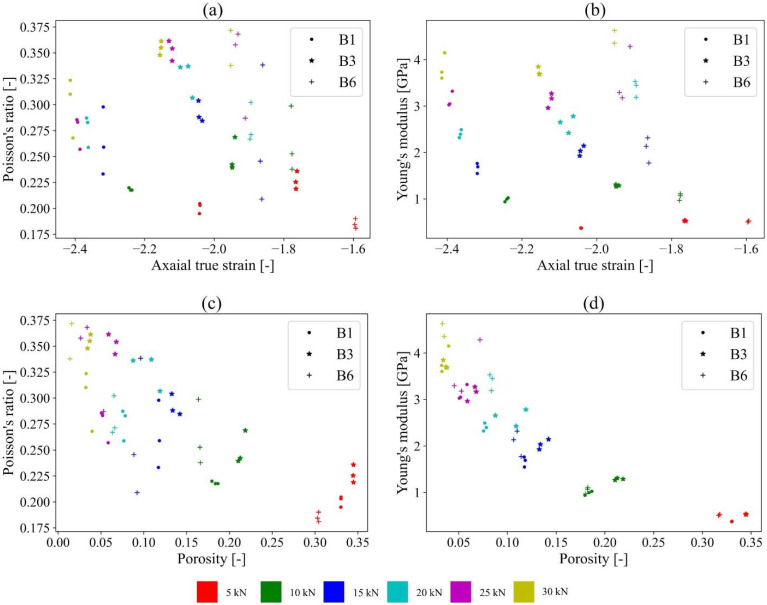
Poisson’s ratio and Young’s modulus of granule batches B1, B3, B6 at different compaction force levels. Poisson’s ratio—axial true strain (**a**); Young’s modulus—axial true strain (**b**); Poisson’s ratio—porosity (**c**); Young’s modulus—porosity (**d**).

The next step aimed at identifying the elastic properties of the paper granules. For this, double compaction tests were carried out for 5–30 kN compaction levels via 5 kN steps. From the results, the respective Poisson’s ratio and Young’s modulus were determined to investigate the influence of compression forces on the elastic properties and the compressibility of the paper granules with different sucrose content (Figure 4). 

The main-compaction loading curves are not linear when low forces and forces greater than the pre-compaction force level are applied to the granules. This can be attributed to the porosity of the material at the beginning of the compression process [23]. Therefore, to determine the elastic constants, only the linear part of the main-compaction loading curves was used for the calculation of the elastic material properties, i.e., Poisson’s ratio and Young’s modulus. The linear part was determined to be within 30–80% compared to the pre-compaction force levels. Young’s modulus represents the stiffness of a material. Hence, the higher the Young’s modulus, the higher is the stiffness of the material. The Poisson’s ratio is defined to be the deformation of a material in directions perpendicular to the specific direction of loading. A high Poisson’s ratio indicates that the material has a high perpendicular deformation, meaning that—under axial force—the material “escapes” due to low compressibility. Data obtained from the double compaction tests (Figure 4a,b) show that, for all batches, both the Poisson’s ratio and the Young’s modulus increase nonlinearly with increasing compaction. Hence, during compaction, the paper granules become more compact and stiffer, which also results in an increased density and reduced porosity (Figure 4c,d). The Poisson’s ratio at higher compaction force levels increased with increasing sucrose content and Young’s modulus remained almost constant for the different sucrose contents at similar compaction force levels (Figure 4a,b). However, the different sucrose contents had no influence on the changes in porosity (Figure 4a,b). Hence, after compression, independent of the sucrose content, similar porosities were obtained.

**Figure 5:**
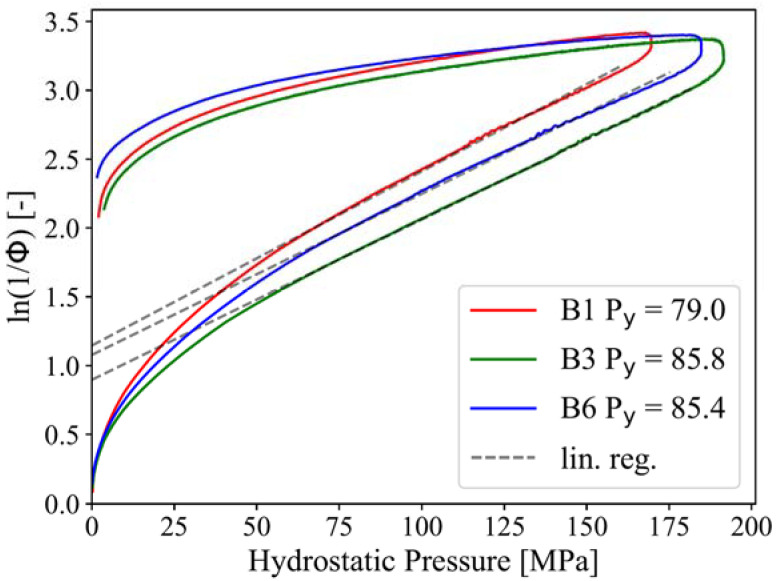
Heckel plots calculated for the three batches and linear regression fit to determine the yield Pressure (P_y). Coefficient of determination for the fits are: R² =0.999 for B1, R² = 0.9999 for B3 and R² = 0.9989 for B6.

In the next step, the data obtained were used to judge if paper granules possess sufficient binding properties rendering them into suitable intermediate products for the production of tablets in large, industrial scale. Binders or compression enhancers with excellent binding properties should be made from soft materials that undergo plastic deformation during compaction [24]. Examples for this are microcrystalline cellulose and various cellulose ethers [20,24]. Today, in many cases, the Heckel mathematical model is used to judge the compressibility of a material [24]. The Heckel equation describes the relationship between the process relating porosity (φ) and the hydrostatic pressure (*P*) (cf. 3.2.4.6.) and allows us to determine the so-called yield pressure (*P*_y_, cf. formula 8), which describes the necessary pressure to plastically deform a material. Low yield pressures represent a soft/plastic behavior, whereas higher values indicate a hard/brittle and behavior [20]. The yield pressures of the paper granules ranged from 79–86 MPa (Figure 5). With this they are in the upper limit (80 MPa) to be classified as soft/plastic material, indicating a very good compressibility of the paper granules [20]. However, they are also close to the lower limit of a hard/brittle behavior. The trend towards a higher hardness and a more brittle behavior can especially be seen for the batches with higher sucrose content. 

This is reasonable as sucrose is a crystalline and brittle material [25]. Hence, higher contents of sucrose within the paper-based granules results in a higher proportion of crystalline and brittle material within the granules, which should then result in a higher cracking propensity [20]. However, the trend is not significant. This means that it can be summarized that the sucrose content had no significant influence on the yield pressure. Moreover, it can be stated that the deformation behavior of paper granules—independent on the sucrose content—is comparable to other pharmaceutical binders. In fact, paper granules were found to be suitable intermediate products for the production of tablets in large, industrial scale. The material properties of the paper granules are also sufficient for yielding tablets that fulfill the criteria according to the European Pharmacopeia. 

In addition the data acquired here also provide the base for the establishment of numeric models that are able to simulate the compression process of powder into tablets. Different methods are available for this, i.e., the Discrete Element Method (DEM) [26] and the Finite Element Method (FEM) [27]. FEM is more commonly used to simulate powder compression as it represents the powder as an isotropic continuous media [28,29,30,31]. One of the most commonly used models in FEM is the Drucker-Prager Cap plasticity model (DPC) [32,33] and it has been previously utilized for compression simulations for the production of tablets with optimal pharmaceutical properties [28,29,30,31]. Such modeling allows us, for example, to predict the shape and the size of the resulting tablets. It also enables us to predict inhomogeneous properties within the tablets which can result in insufficient pharmaceutical quality of the tablets (for example capping of the tablets). 

The understanding and modulation of such processes thus allows for the development of efficient compression processes that result in tablets with optimal physico-chemical and pharmaceutical properties. Such simulations can also predict the hardness of tablets, which then provides a close link to the disintegration of the tablet and with this also a close link to the dissolution profile of the active ingredient out of the tablet. Future work should now use the data acquired here to establish simulation models that help to predict the above-mentioned parameters. These parameters should then be linked to the biopharmaceutical properties, i.e., bioavailability and pharmacokinetic profiles. The link between simulation and biopharmaceutical performance would then allow for a fast and efficient development of drug-loaded smartFilm tablets with optimal pharmaceutical properties. 

## 3. Materials and Methods

### 3.1. Materials

A commercially available, cellulose-based paper (Soft & Sicher, dm-drogerie markt GmbH + Co. KG, Karlsruhe, Germany) was used to produce the granules and tablets. Sucrose was purchased from Carl Roth GmbH + Co. KG (Karlsruhe, Germany). Purified water was freshly obtained from a PURELAB Flex 2 (ELGA LabWater, Veolia Water Technologies GmbH, Celle, Germany).

### 3.2. Methods

#### 3.2.1. Production of Paper Granules

Paper granules were prepared via the wet granulation method. Distilled water or sucrose aqueous solutions with various concentrations (10, 20, 30, 40 and 50% *w*/*w*) were used as a granulation liquid. To prepare paper granules, a paper sheet that weighted around 2.50 g, was dry milled for 1 min by using a knife mill (Moulinex DP8108, Groupe SEB Deutschland GmbH, Frankfurt, Germany). The milled paper was used to prepare 11 granule batches coded as B1–B11 (Table 1). B1 was the control, i.e., paper granules without sucrose. For the batches B2–B6 sucrose (10%, 20%, 30%, 40% and 50%) was used as dry binder and for the batches B7–B11 sucrose (10%, 20%, 30%, 40% and 50%) was used as liquid binder. B1 was prepared by spraying small amounts of water onto the milled paper to wet the paper and to increase its density. The wetted mixture was again milled for 1 min, transferred to a plastic sieve, wetted with distilled water to obtain snowball consistency while shaking at 300 rpm in a universal shaker SM-30 control (Edmund Bühler GmbH, Bodelshausen, Germany) for a period of 3–8 min. The wet granules were dried in a drying oven (Heraeus GmbH, Hanau, Germany) at 70 °C overnight. Afterwards, the granules were sieved (mesh size 2.8 mm, Retsch GmbH, Haan, Germany) to obtain a size fraction ≤ 2.8 mm. B2–B6 were produced by adding fine sucrose powder to the dry milled paper to obtain dry paper/sucrose mixtures that contained 10%, 20%, 30%, 40% and 50% *(w/w)* sucrose. The mixtures were further processed as described for B1. B7–B11 were produced by adding sucrose aqueous solutions to the dry milled paper to obtain mixtures that contain 10%, 20%, 30%, 40% and 50% (*w*/*w*) sucrose, respectively. Afterwards, they were processed as described for B1, while using the respective sucrose solutions instead of purified water. 

#### 3.2.2. Characterization of Paper Granules

##### Particle Size and Shape Analysis 

The Feret’s diameter of the granules was determined via digital image analysis using ImageJ software (National Institutes of Health, Bethesda, MD, USA) as described previously [34], with slight modifications. For each batch, 10 representative images that contained approximately 200–300 granules were taken by using a Canon IXUS 190 digital camera (Canon Europe Ltd., Uxbridge, UK). The images were color-adjusted, and threshold analysis was performed to label the granules individually. Afterwards, the Feret’s diameter of each granule was assessed by the software (Supplementary Material, Table S1). From the results obtained the number based median particle size distribution, i.e., d(n) 0.10, d(n) 0.50, d(n) 0.90, d(n) 0.95 and d(n) 0.99, was calculated. In addition, the sphericity of the granules was determined via calculating the aspect ratio as follows:

(1) $$\mathrm{Aspect}\;\mathrm{ratio}=\frac{d_{\max\left(\mathrm{Feret}\right)}}{d_{90{}^{\circ}\left(\mathrm{dmax}\right)}}$$

where *d*_max_ (Feret) is the maximum Feret’s diameter and *d*90°(*d*_max_) is the Feret’s diameter perpendicular to *d*_max_ (Feret).

##### Determination of Bulk and Tapped Density

A mechanical tapping device, the tap density tester TD200 (Pharma Test Apparatebau AG, Hainburg, Germany), was used to determine the bulk and tapped density of various batches of the granules according to the test method 2.9.34 of the European Pharmacopeia [10]. A total of 10 g of the granules were placed into a 250 mL measuring cylinder. The starting volume and the final volume, after carrying out 10, 500 and 1250 taps on the same granules sample, were recorded and used to calculate the bulk and tapped density, respectively. In addition, the flowability of the granules was determined from the tapped and bulk density by calculating Hausner’s ratio and the Carr’s index using the following equations [35]:

(2) $$Hausner’s\;ratio=\frac{\rho_{\mathrm{tapped}}}{\rho_{\mathrm{bulk}}}$$

(3) $$Carr’s\;index=100\left(\frac{\rho_{\mathrm{tapped}-}\rho_{\mathrm{bulk}}}{\rho_{\mathrm{tapped}}}\right)$$

where ρ_tapped_ is the tapped density and ρ_bulk_ is the bulk density.

##### Determination of Angle of Repose 

The angle of repose of the granules was determined by using the flowability tester (Karg GmbH, Baden-Württemberg, Germany) according to the test method 2.9.36 of the European Pharmacopeia [10]. A total of 20 g of the granules from various batches were filled into the funnel of the tester. Then, the granules were stirred carefully to run through the funnel and accumulate on a fixed base to form a heap. The height of the granules heap was measured and the angle of repose (α) was determined using the following equation:

(4) $$\tan\alpha =\frac{h}{0.5\;d_{b}}$$

where *h* is the height of the heap and *d*_b_ is the diameter of the base.

#### 3.2.3. Production of Tablets Made from Paper

Flat-faced bevel-edged tablets were produced by applying compression forces of about 30 kN by using a single punch tablet press (EK0, Korsch GmbH, Berlin, Germany) equipped with a 10 mm flat-faced punch (Ritter Pharma-Technik GmbH, Stapelfeld, Germany). Subsequently, the properties of the produced tablets were assessed by subjecting the tablets to various tests, as described in the European Pharmacopeia [10]. Details of the methods used are given below.

#### 3.2.4. Characterization of Tablets Made from Paper

##### Thickness

A total of 10 tablets were selected randomly from each batch and the thickness was determined using IP67 ABS digimatic caliper (Mitutoyo, Kanagawa, Japan). 

##### Mass Uniformity

Mass uniformity was evaluated according to the test method 2.9.5. of the European Pharmacopeia [10]. A total of 20 tablets were randomly selected from each batch and weighed. Then, the average mass was calculated and compared to the mass of each individual tablet to determine the percentage deviation; following this, the results were compared to the European Pharmacopeia limits.

##### Friability 

Friability of the tablets was determined according to test method 2.9.7 of the European Pharmacopeia [10] by using a friability tester equipped with an abrasion drum (PTF 10ER, Pharma Test Apparatebau AG, Hainburg, Germany). From each batch, 20 tablets were randomly selected, weighed, then placed into a drum rotating at 25 rpm for 4 min. Following that, the tablets were removed, dedusted, reweighed and the percentage weight loss was calculated using the following equation:

(5) $$\%\;\mathrm{weight}\;\mathrm{loss}=100\left(\frac{W_{1}-W_{2}}{W_{1}}\right)$$

where *W*_1_ is the weight of the tablets before test and *W*_2_ is the weight of the tablets after test.

##### Resistance to Crushing 

The crushing strength or hardness of a tablet is the force required to break down a tablet under compression and it was evaluated according to the test method 2.9.8. of the European Pharmacopeia [10]. In this study, 10 tablets were randomly selected from each batch and each tablet was placed horizontally between the jaws of a hardness tester PTB 311E (Pharma Test Apparatebau AG, Hainburg, Germany). The results obtained were expressed in Newton (N) as mean, minimum and maximum values of the forces measured. 

##### Disintegration

Disintegration of the tablets was evaluated in water according the test method 2.9.1 of the European Pharmacopeia [10]. From each batch, 6 tablets were individually placed into the cavities of a disintegration tester PTZ-S (Pharma Test Apparatebau AG, Hainburg, Germany) that was operated at 37 °C ± 2 °C. The time required for complete tablet disintegration was recorded and compared to the limits of the European Pharmacopeia [10].

##### Mechanical Behavior of Paper Granules under Compression

The mechanical characterization was performed by using the single punch tableting press (Styl’One Evo, Medelpharm, Beynost, France) via displacement control. Axial forces of the upper and lower punch were measured using strain gauges and axial displacements were determined by a potentiometric displacement transducer. The global axial elastic deformation of the machine was considered when evaluating the measurement data of the tests. Radial stress components on the die wall were measured using strain gauges attached to the die. Calibration of the instrumented die was conducted by the compression of a nearly incompressible elastomer.

Standard Euro B flat-faced punches with a diameter of 11.28 mm were used for the tests. The maximum possible filling height of the die was 23.5 mm and 250 mg of paper granules were manually filled into the die for each compression test. To determine the strain rate effects, experiments were carried out at low (0.1 mm/s) and high (500 mm/s) displacement velocities. Die wall friction (µ) was calculated during the instrumented die tests using Coulomb’s friction law:

(6) $$µ=\frac{D\left(\sigma_{up}-\sigma_{lo}\right)}{4h\sigma_{rad}}$$

where *D* is the diameter of the die, *h* is the tablet height, *σ_up_* and *σ_lo_* are the upper and lower punch stress, respectively, and σ_rad_ is the radial die wall stress [30]

Double-compaction tests were carried out to identify Young’s modulus and Poisson’s ratio at different compaction force levels [23]. The granules were pre-compacted, unloaded and directly followed by a main compaction (i.e., no ejection of the produced tablets during the entire process). The pre-compaction maximum force was set to 90% of the desired main-compaction force. During the loading of the main-compaction, linear elasticity was assumed when the forces remain lower than the maximum pre-compaction force and allows for linear stress—strain relations. A detailed description of the process and the determination of the constants is described in Mazel et al., 2012 [12]. The porosity at the different stress levels, at which the values of the elastic constants (i.e., Young’s modulus and Poisson’s ratio) were determined, was calculated. This allows for the interpolation of the elastic constant values with porosity during the compression. The true density ρ_0_ was determined before the tests by a gas pycnometer (AccuPyc II 1340, Micromeritics, Norcross, GA, USA) and was used to calculate the porosity of the materials (ϕ), where ρ is the actual density.

(7) $$\phi =1-\frac{\rho }{\rho_{0}}$$

Finally, the Heckel Plot was used to determine the yield pressure, as it is widely used for the characterization of the compression and deformation behavior of powders.

(8) $$\ln\left(\frac{1}{\phi }\right)=kP+A$$

where constant *k* is the slope of the linear plastic deformation section and *A* is the intercept and is suggested to represent particle rearrangement. The reciprocal of the *k* is the mean yield pressure $P_{y}$ defining the pressure at which the material undergoes plastic deformation. The Heckel plot is used to describe the volume reduction of the paper granules during compression, as the porosity is proportional to hydrostatic pressure *P* (i.e., compression pressure).

(9) $$P=\frac{\sigma_{\mathrm{ax}}+\sigma_{\mathrm{rad}}+\sigma_{\mathrm{rad}}\;}{3}$$

With axial stress $\sigma_{ax}$ and radial stress $\sigma_{\mathrm{rad}}$ measured during the instrumented die test.

#### 3.2.5. Statistical Analysis

Experiments were performed in triplicates unless otherwise noted and descriptive statistics were calculated using Microsoft Excel^®^ and are reported as mean ± standard deviation (SD). Normal distribution (Shapiro-Wilk test) and variance homogeneity (Levene‘s test) were determined for each data set prior to statistical assessment of differences between the granule batches. The comparison of the means was performed by two-sided Student’s *t*-test for pairwise comparison or one-way analysis of variance and post-hoc tests with Tukey correction using JASP software, version 0.16.2 [36]. Differences between means were considered statistically significant if the *p* value was <0.05.

## 4. Conclusions

The aim of the study was to transform paper into a flowable physical form and to investigate the influence of sucrose on the resulting material properties. Paper was successfully transformed into paper granules via a wet granulation process. The resulting granules had a size of about 3 mm with a slightly elongated shape. The sucrose granules possessed good flowability and allowed for the production of tablets in continuous, i.e., high-speed, tableting mode. The addition of sucrose as a dry powder was found to be the most suitable production process for the paper granules. The tablets made of these paper granules possessed pharmaceutical properties according to the European Pharmacopeia. The deformation behavior of the paper granules was determined and compared to the properties that are required for excipients that are used as binders and/or compression enhancers. Based on the results, it can be concluded that paper granules possess a suitable compression behavior, thus rendering them suitable intermediate products for the production of tablets made from paper on a larger, industrial scale. 

## Figures and Tables

**Table 1:** Overview of compositions of the different batches of paper granules produced.

Batch Code	Sucrose Content (Dry Form)	Granulation Liquid
B1	-	distilled water
B2	10%	distilled water
B3	20%	distilled water
B4	30%	distilled water
B5	40%	distilled water
B6	50%	distilled water
B7	-	10% sucrose solution
B8	-	20% sucrose solution
B9	-	30% sucrose solution
B10	-	40% sucrose solution
B11	-	50% sucrose solution

**Table 2:** Physico-chemical properties of the different paper granules produced in this study.

Batch Code	Feret’s Diameter (mm)	Bulk Density (g/cm^3^)	Tapped Density (g/cm^3^)	Hausner’s Ratio	Carr’s Index (%)	Angle of Repose
B1	3.0 ± 1.2	0.11 ± 0.003	0.13 ± 0.003	1.12 ± 0.01	11.4 ± 0.4	43° ± 4
B2	3.4 ± 0.67	0.12 ± 0.001	0.14 ± 0.001	1.15 ± 0.00	13.3 ± 0.1	34° ± 4
B3	3.3 ± 0.85	0.14 ± 0.003	0.15 ± 0.002	1.13 ± 0.04	11.7 ± 3.6	31° ± 0
B4	3.2 ± 0.79	0.14 ± 0.01	0.17 ± 0.01	1.16 ± 0.02	13.8 ± 2.0	31° ± 0
B5	3.4 ± 0.83	0.17 ± 0.008	0.20 ± 0.006	1.13 ± 0.00	11.4 ± 0.5	25° ± 5
B6	3.3 ± 0.89	0.21 ± 0.005	0.22 ± 0.007	1.13 ± 0.03	11.9 ± 2.8	25° ± 5
B7	3.3 ± 0.90	0.15 ± 0.015	0.18 ± 0.017	1.14 ± 0.00	13.0 ± 0.0	34° ± 4
B8	3.3 ± 0.88	0.16 ± 0.004	0.18 ± 0.004	1.15 ± 0.01	13.1 ± 0.9	31° ± 0
B9	3.0 ± 0.86	0.18 ± 0.001	0.20 ± 0.005	1.13 ± 0.03	11.8 ± 2.3	31° ± 0
B10	3.55 ± 0.98	0.20 ± 0.017	0.22 ± 0.014	1.13 ± 0.03	11.7 ± 2.3	25° ± 5
B11	3.41 ± 6.75	0.17 ± 0.016	0.20 ± 0.014	1.15 ± 0.02	13.4 ± 1.8	25° ± 5

**Table 3:** Physico-chemical properties of the different paper granules produced in this study.

Batch Code	Thickness (mm)	Mass Uniformity (%)	Friability (%)	Hardness (N)	Disintegration
B1	1.8 ± 0.01	4.9 ± 2.8	<0.001	20.8 ± 7.6 min.: 10.8 max.: 32.6	all tablets disintegrated within 10 s
B2	1.3 ± 0.02	1.8 ± 1.6	0.14	51.5 ± 12.9 min.: 35.2 max.: 71.5	all tablets disintegrated within 2 min
B3	1.7 ± 0.04	1.8 ± 1.6	0.23	112.8 ± 18.6 min.: 84.2 max.: 129.5	all tablets disintegrated within 5 min
B4	1.8 ± 0.02	3.1 ± 1.8	0.03	123.4 ± 22.2 min.: 97 max.: 142	all tablets disintegrated within 15 min
B5	2.0 ± 0.04	3.2 ± 2.9	0.09	154.2 ± 15.3 min.: 127.1 max.: 173.3	all tablets disintegrated within 35 min
B6	3.3 ± 0.18	4.0 ± 2.9	0.10	250.3 ± 24.6 min.: 210.2 max.: 279	all tablets disintegrated within 50 min
B7	1.4 ± 0.03	3.1 ± 1.9	0.11	71.1 ± 14.6 min.: 52.5 max.: 99.9	all tablets disintegrated within 20 min
B8	1.8 ± 0.04	2.4 ± 2.1	0.04	166.1 ± 29.3 min.: 116.1 max.: 217.2	all tablets disintegrated within 20 min
B9	1.9 ± 0.06	3.8 ± 2.3	0.01	221.7 ± 60.4 min.: 122.9 max.: 288.2	all tablets disintegrated within 45 min
B10	2.0 ± 0.04	2.6 ± 2.1	0.14	258.6 ± 28 min.: 200.9 max.: 290.7	all tablets disintegrated within 55 min
B11	2.0 ± 0.07	4.6 ± 2.3	0.01	271.5 ± 29.2 min.: 221.9 max.: 300	all tablets disintegrated within 60 min

## Data Availability

Data is included within the article and supplementary material.

## Supplementary Materials

The following supporting information can be downloaded at: https://www.mdpi.com/article/10.3390/ph15101188/s1, Table S1: Macro used for the determination of the Feret’s diameter.
