# Supplementary material for: Tablets Made from Paper—An Industrially Feasible Approach
Source: Pharmaceuticals (Basel). 2022 Sep 26;15(10):1188. doi: 10.3390/ph15101188 (PMC9611322; doi:10.3390/ph15101188)
Supplement: Supplementary file 1 [file pharmaceuticals-15-01188-s001.zip › pharmaceuticals-1880326-supplementary.pdf]

pharmaceuticals

Article

Tablets made from paper - an industrially feasible approach

Ayat Abdelkader [1,2,], Christoph Moos [3,**], Adrien Pelloux [4], Marcus Pfeiffer [3], Christian Alter [3], Stefan Kolling [3] and Cornelia M. Keck [1,*]**

1 Department of Pharmaceutics and Biopharmaceutics, Philipps-Universität Marburg, Robert-Koch-Str. 4, 35037 Marburg, Germany
2 Assiut International Center of Nanomedicine, Al-Rajhy Liver Hospital, Assiut University, 71515 Assiut, Egypt
3 Institute of Mechanics and Materials, Technische Hochschule Mittelhessen, Wiesenstr. 14, 35390 Giessen, Germany
4 MEDELPHARM, Science Lab, rue du Chat Botté 615, 01700 Beynost, France

* Correspondence: e-mail@e-mail.com; Tel.: (optional; include country code; if there are multiple corresponding authors, add author initials)

** contributed equally

Supplementary Materials:

Table S1: Macro used for the determination of the Feret`s diameter.

```
run("8-bit");
setAutoThreshold("Default");
//run("Threshold...");
//setThreshold(0, 180);
setOption("BlackBackground", false);
run("Convert to Mask");

run("Analyze Particles...", "size=0.09-Infinity show=Masks display");
```
